# Direct and indirect effects of multiplex genome editing of F5H and FAD2 in oil crop camelina

**DOI:** 10.1111/pbi.14593

**Published:** 2025-01-27

**Authors:** Jarst van Belle, Jan G. Schaart, Annemarie C. Dechesne, Danli Fei, Abraham Ontiveros Cisneros, Michele Serafini, Richard G.F. Visser, Eibertus N. van Loo

**Affiliations:** ^1^ Wageningen University and Research Plant Breeding Wageningen The Netherlands; ^2^ Linnaeus Plant Sciences B.V. Wageningen The Netherlands; ^3^ HAN University of Applied Sciences, Applied Biosciences and Chemistry Nijmegen The Netherlands

**Keywords:** camelina, seed oil, ferulate 5‐hydroxylase, fatty acid desaturase 2, sinapine, syringyl monolignol

## Abstract

Mutants with simultaneous germline mutations were obtained in all three *F5H* genes and all three *FAD2* genes (one to eleven mutated alleles) in order to improve the feed value of the seed meal and the fatty acid composition of the seed oil. In mutants with multiple mutated *F5H* alleles, sinapine in seed meal was reduced by up to 100%, accompanied by a sharp reduction in the S‐monolignol content of lignin without causing lodging or stem break. A lower S‐lignin monomer content in stems can contribute to improved stem degradability allowing new uses of stems. Mutants in all six *FAD2* alleles showed an expected increase in MUFA from 8.7% to 74% and a reduction in PUFA from 53% to 13% in the fatty acids in seed oil. Remarkably, some full *FAD2* mutants showed normal growth and seed production and not the dwarfing phenotype reported in previous studies. The relation between germline mutation allele dosage and phenotype was influenced by the still ongoing activity of the CRISPR/Cas9 system, leading to new somatic mutations in the leaves of flowering plants. The correlations between the total mutation frequency (germline plus new somatic mutations) for *F5H* with sinapine content, and *FAD2* with fatty acid composition were higher than the correlations between germline mutation count and phenotypes. This shows the importance of quantifying both the germline mutations and somatic mutations when studying CRISPR/Cas9 effects in situations where the CRISPR/Cas9 system is not yet segregated out.

## Introduction

Camelina (*Camelina sativa* (L.) Crantz) is an emerging oilseed crop for biofuels and bio‐based materials because of its broad tolerance to (a)biotic stress and minimal agronomic input requirements compared to established oilseed crops (soybean, sunflower and rapeseed) (Iskandarov *et al*., 2014). Globally, over 40 000 ha of camelina is grown (e.g. in Canada more than 10 000 ha) and the acreage is increasing, because the short growth cycle of camelina enables use in areas where other crops cannot or no longer reach maturity in time.

Camelina oil is used both as fish feed and towards production of biodiesel. The seed meal with around 45% of high‐quality proteins can be used for livestock feed (Das *et al*., 2014; Korsrud *et al*., 1978; Righini *et al*., 2016) but only to a limited extent among others due to the presence of bitter sinapine (Riaz *et al*., 2022). Sinapine also reduces protein digestibility (Shahidi and Naczk, 1992). Camelina seed meal could even be used as a new source of plant protein for human food products if the level of anti‐nutritional factors were sharply reduced (Goh *et al*., 1979; Iskandarov *et al*., 2014; Matthäus and Angelini, 2005; Righini *et al*., 2016).

The seeds of camelina contain 30%–40% oil with an oil composition that is high in poly‐unsaturated fatty acids (FA) with a high proportion of C18:3 (linolenic, 32%), C18:2 (linoleic, 21%) and moderate proportions of C18:1 (oleic, 15%), C20:1 (gondoic, 12%) and a low proportion of C22:1 (erucic, <4%) fatty acids (Iskandarov *et al*., 2014; Vollmann *et al*., 1996).

C18:3 and C18:2 are essential omega‐3 and omega‐6 polyunsaturated FA (PUFA), while C18:1, C20:1 and C22:1 are monounsaturated FA (MUFA) that can be chemically converted into high‐value C10:0, C12:0 and C14:0 (capric, lauric and myristic) FA as medium‐chain FA (MCFA) sources for polymer building blocks for the production of polyamides such as nylons, for example, using new processes like olefin metathesis (Chikkali and Mecking, 2012; Couturier and Dubois, 2014; Zubr, 1997). Although the oleochemical industry has identified camelina oil as an interesting feedstock, the high contents of PUFA and too low contents of MUFA currently make it economically unfeasible. This underscores the need to improve both the quality of the seed meal and the composition of the seed oil.

In transgenic approaches for modification of seed meal and seed oil quality, RNAi has been used to silence the *FAD2* gene, resulting in a changed fatty acid composition (Horn *et al*., 2013; Nguyen *et al*., 2013). The introduction of non‐camelina genes into camelina has been reported to achieve the accumulation of 4‐vinyl phenols by expression of a bacterial gene (phenolic acid decarboxylase, PAD) in camelina (Menard et al. 2022). This led to a 95% reduction of sinapine content in the seeds of camelina. An alternative strategy where the final product does not contain transgenic elements can offer advantages because of a lower regulatory burden (e.g. in the EU, policies are directed at removing the need for labeling and allowing easier release of gene edited products, for example, produced with CRISPR/Cas9).

Also, EMS mutation strategies have been developed in camelina (Neumann *et al*., 2021), but as per EMS mutant usually only one locus is mutated, they have the disadvantage that targeting many loci will require many generations of crossing to combine all mutations.

This paper uses a multiplex CRISPR/Cas9 approach (Lowder *et al*., 2015; Ma *et al*., 2015) to achieve mutations to reduce the level of antinutritional factors (ANF) in seed meal (specifically sinapine) and to improve the composition of the seed oil simultaneously. While multiplex CRISPR/Cas9 strategies have been utilized in camelina to modify flowering and architecture traits (Bellec *et al*., 2022), multiplex CRISPR/Cas9 approaches for targeting genes involved in seed meal quality and seed oil quality have not been reported. We have achieved this by targeting *F5H*, a gene coding for ferulate 5‐hydroxylase which catalyzes the production of precursors of sinapine (KEGG, 2019) and separately and simultaneously targeting *FAD2*, which is a gene coding for fatty acid desaturase 2, a delta‐12 desaturase creating an extra double bond at the n‐6 position in MUFA, thereby catalyzing the production of C18:2 from C18:1 fatty acid (Hutcheon *et al*., 2010).

Recent studies have already achieved *FAD2* knockouts in camelina with improved FA profiles, but full knock‐outs of all *FAD2* genes in camelina resulted always in dwarfism (reduced internode elongation) as a pleiotropic effect (Jiang *et al*., 2017; Morineau *et al*., 2017). *F5H* knockouts and *F5H* up‐ or downregulation have been described in *Arabidopsis* and a few other species (tobacco, poplar, miscanthus, switchgrass, sugar cane, rapeseed), but the effects on sinapine levels in seeds were not studied (Cao *et al*., 2022; Franke *et al*., 2000; Golfier *et al*., 2021; Huntley *et al*., 2003; Portilla Llerena *et al*., 2024; Shafiei *et al*., 2023; Wu *et al*., 2019). *F5H* knockouts with improved sinapine or lignin profiles have not been described before in camelina.

In this paper, we report on the fully reduced sinapine content in the seed and seed meal in CRISPR mutants with simultaneous knockouts of all three loci of *F5H* and/or *FAD2* in hexaploid camelina using a single CRISPR/Cas9 event with four single guide RNAs (sgRNAs) targeting either *F5H* or *FAD2* or two sgRNAs for each gene to knock out all six loci of both genes simultaneously. We describe camelina lines with mutations in one to three loci of *F5H* and *FAD2*. These mutants show reduced PUFA and enriched MUFA contents in the seed oil, in the absence of dwarfism as a pleiotropic effect. Further, we show that camelina *F5H* mutants with no or reduced sinapine also have a lower syringyl monolignol content in the lignin of stems (Cao *et al*., 2022; Huntley *et al*., 2003). Ultimately, we obtained normally developing camelina mutant plant lines with improved seed oil quality (less PUFA for improved use in oleochemistry), improved feed quality (almost no sinapine) and a lignin composition of stems more suitable for producing biofuels or use as feed. As the CRISPR/Cas9 system was not yet segregated out, both the germline mutation frequency (inherited from the T1 plant to the T2 generation) and the frequencies of additional somatic mutations were determined in order to relate mutant allele dosage effects to the quantitative change in phenotypes.

## Materials and Methods

### CRISPR/Cas9 construct design and assembly

Different CRISPR/Cas9 binary vector constructs were designed and assembled to generate *Camelina sativa* CRISPR/Cas9‐mutants and to evaluate mutation efficiency. *Arabidopsis thaliana* codon‐optimized *Cas9* (*AtCas9*) was expressed under three different promoters: (1) *pdCaMV35S* (double cauliflower mosaic virus 35S promoter), (2) *pPcUbi4‐2*, the *Petroselinum crispum* ubiquitin 4‐2 promoter, and (3) *pRPS5A*, the *A. thaliana* ribosomal protein subunit 5A promoter (Engler *et al*., 2014; Fauser *et al*., 2014; Kawalleck *et al*., 1993; Kay *et al*., 1987; Lampropoulos *et al*., 2013; Weijers *et al*., 2001). The sgRNAs containing the target‐specific spacer sequences were expressed under *pU6‐26* (and *pU6‐1* in an earlier experiment) which is a promoter for the snRNA gene U6 of the *A. thaliana* (Lawrenson *et al*., 2015; Li *et al*., 2007, 2013, 2014; Waibel and Filipowicz, 1990). Selection markers *pCVMV::DsRed::tMas* (red fluorescence) and *pNos::NptII::tOcs* (kanamycin antibiotic resistance) were included in the binary vector constructs for the selection of camelina transformants (Baird *et al*., 2000; Beck *et al*., 1982). The sgRNAs were designed to target 5′‐proximal conserved coding sequences of the three different *FAD2* loci in camelina *FAD2‐A* (alias *FAD2‐2*, LOC104776214, chromosome 1), *FAD2‐B* (alias *FAD2‐3*, LOC104745425, chromosome 15) and *FAD2‐C* (alias *FAD2‐1*, LOC104764975, chromosome 19) and the three *F5H* loci *F5H‐A* (LOC104716504 on chromosome 10), *F5H‐B* (LOC104721146 on chromosome 11) and *F5H‐C* (LOC104729559 on chromosome 12) (Hutcheon *et al*., 2010), using assembly GCF_000633955.1 (genome sequence of cultivar DH55). Naming conventions used on NCBI have been used. Supplemental information Data S1 contains the genomic sequences of these loci as reported for DH55. In cultivar Cypress, a few SNPs occur compared to DH55 in these genes, and in *FAD2‐B* a natural 3 nt insertion is present close to the start of the gene.

**Figure 1:**
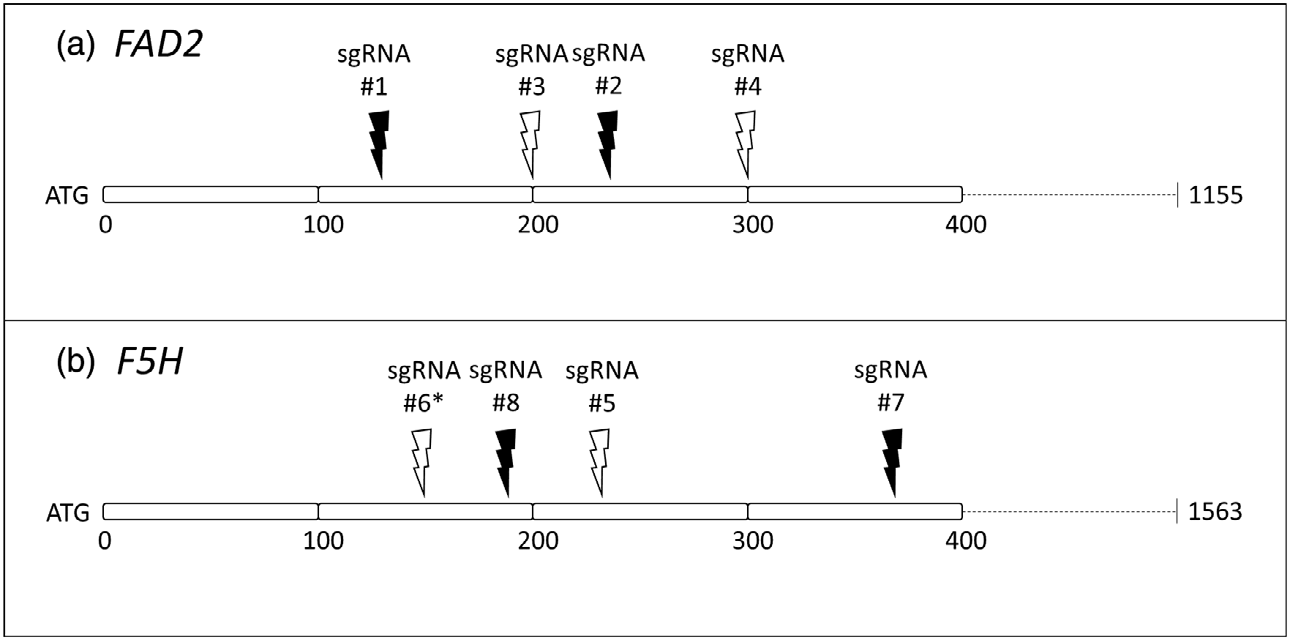
Schematic representation of the first 400 bp of the *FAD2* (a) and *F5H* (b) coding sequences with target sites of the designed sgRNAs (Table S1.1) with their relative positions compared to the ATG start codons in *Camelina sativa* cv. Cypress. sgRNAs #1/#2/#3/#4 were combined in L2‐vectors to target *FAD2*, sgRNAs #5/#6/#7/#8 to target *F5H*, and sgRNAs #1/#2/#7/#8 (indicated in black) to simultaneously target *FAD2* and *F5H*. *: incorrectly designed sgRNA.

The sgRNAs were designed with the WU‐CRISPR tool (Xiaowei Wang Lab, Washington University, St. Louis, MO, USA), which also predicts the sgRNA efficiency by, among others, calculating the sgRNA secondary structure (Doench *et al*., 2014; Hofacker, 2003; Wong *et al*., 2015). A single G‐nucleotide was added to the 5′‐end of each 19‐nucleotide‐long sgRNA spacer sequence to initiate sgRNA expression under *U6* promoters and increase overall sgRNA activity (Moreno‐Mateos *et al*., 2015; Sander and Joung, 2014). Further, each sgRNA was evaluated for potential off‐target CRISPR/Cas9 specificity in *C. sativa* with the BLASTN tool (NCBI). Table S1.1 shows an overview of the *FAD2* and *F5H* gene‐specific sgRNAs. One sgRNA was incorrectly designed by unintendingly adding a single nucleotide between the sgRNA target site and PAM and was also included to evaluate the performance of CRISPR/Cas9 systems. CRISPR/Cas9 constructs were assembled using the Golden Gate cloning system (Weber *et al*., 2011). This cloning system involved the subsequent cloning of level 0 (L0), level 1 (L1) and level 2 (L2) vectors. L1 vectors, containing the expression cassettes of the sgRNAs and *AtCas9* and selection markers *pCVMV::DsRed::tMas* and *pNos::NptII::tOcs*, were combined into an L2 end vector (pICSL4723, i.e. a fixed version of pAGM4723; Addgene Plasmid #86173) (Baird *et al*., 2000; Beck *et al*., 1982). The L1 sgRNA cassettes included *pU6‐26* from pICSL90002 (Addgene Plasmid #68261), or *pU6‐1* from pUC119‐gRNA (Addgene Plasmid #52255) in earlier experiments of this study (Lawrenson *et al*., 2015; J.‐F. Li *et al*., 2013). The L1 *Cas9* cassettes included *AtCas9* from pDe‐CAS9 (Addgene Plasmid #61433), and either *pdCaMV35S* from pICH51288 (Addgene Plasmid #50269), *pPcUbi4‐2* from pDe‐CAS9 (Addgene Plasmid #61433), or *pRPS5A* from pGGA012 (Addgene Plasmid #48818) (Engler *et al*., 2014; Fauser *et al*., 2014; Lampropoulos *et al*., 2013). The designed CRISPR/Cas9 systems in the final L2‐vectors, with different promoters for *AtCas9* and different combinations of sgRNAs targeting *FAD2* and/or *F5H* (Figure 1), were introduced in *Agrobacterium tumefaciens* strain GV3101 by electroporation. CRISPR/Cas9 systems with *pU6‐1::sgRNA* from early experiments are discussed further in the results section. The effectiveness of different promoter‐*Cas9* combinations was compared using non‐parametric Kruskall‐Wallis tests.

### Camelina transformation and transformant selection

Camelina plants of the cultivar Cypress were transformed based on the floral dip methods (Lu and Kang, 2008). At least 24 plants were transformed for each CRISPR/Cas9 construct with four sgRNAs targeting either *FAD2* or *F5H* and 48 plants for constructs with two sgRNAs targeting *FAD2* and two sgRNAs targeting *F5H*.

Floral buds were submerged into an *A. tumefaciens* floral dip suspension with 5% (w/v) sucrose, 1/2 strength MS including Gamborg B5 vitamins (M0231, Duchefa Biochemie, Haarlem, the Netherlands) and 0.05% (v/v) Silwet L‐77 (Van Meeuwen, Weesp, the Netherlands) with OD_600_ values of ~0.6, and incubated for 5 min at −85 kPa using a high‐capacity Secador Techni‐Dome 360 Vacuum Desiccator (F42029‐0000, Bel‐Art, Pocomoke City, MD, USA) and Laboport Vacuum Pump (N840.3FT.18, KNF Neuberger, Freiburg, Germany) in the greenhouse.

Transgenic seeds (T1 seeds) from the dipped plants were selected by DsRed fluorescence using a UV fluorescence stereomicroscope. Leaves positioned close to the flowers of the T1 plants were sampled for mutation detection. Mutations found in these leaves are good indicators of the probability that the gametes of the T1 plants will carry mutations. Leaves from 2 weeks old T2 seedlings of selected T1 plants were sampled to find heritable mutations (although still new somatic mutations may arise). DNA of the T1 and T2 leaves was extracted for PCR of amplicons of the *FAD2* and *F5H* genes for Illumina MiSeq amplicon sequencing.

### High‐throughput mutation sequencing

Amplicons covering the sgRNA target sites of the T1 and T2 camelina transformants were sequenced with Illumina MiSeq (Illumina MiSeq Preparation Workflow from the 16S Metagenomic Sequencing Library Preparation Guide version #15044223 Rev. B), with adaptations (Figure S1). One crucial adaptation was to include ‘1st Stage PCR’ forward primers (i.e. *FAD2* and *F5H* gene‐specific forward primers with 5′‐overhang adapters to allow for ‘2nd Stage PCR’) with unique barcodes between the adapters and gene‐specific primers (i.e. from 5′ to 3′: ATGACG, CGAGTC, GCATCA, TACTGT, CAGCTA, TCGTAG, CTCCAC, GTGGAT). These form barcodes in which any three successive nucleotides were unique. Including these barcodes allowed to pool up to eight *FAD2* with eight *F5H* amplicon samples. A set of 192 Stage 1 PCR pooled amplicon samples went to ‘2nd Stage PCR’ in which the MiSeq indices were added in the library preparation. Both for *FAD2* and *F5H*, the Stage 1 amplicon PCR amplified the DNA fragments from all three genes present in the genome. So, in each of the 192 Stage 1 PCR pooled samples, the amplicon of 8 samples with each three genes of *FAD2* and 8 samples with each three genes of *F5H* was present, so from 48 amplified loci. The coverage obtained per locus on average was above 1000 reads, so rare (somatic) mutations could be found, and mutation frequencies could be estimated accurately. The *FAD2* and *F5H* gene‐specific primers (without adapters and unique barcodes) are shown in Supplemental information Table S1.2. These primers were designed to amplify all three genomic copies of the genes.

Using this approach 135 T1 and 581 T2 camelina mutant plants from 14 selected T1 mutants and 28 camelina wildtype reference plants were analyzed. In total over 3400 unique PCR amplicons were sequenced from 744 plants, some with 6 amplicons (for both *FAD2* and *F5H*) and some with 3 amplicons for either *FAD2* or *F5H*. A more detailed overview of the T1 and T2 camelina samples is shown in Table 1.

**Table 1:** Overview of samples from camelina floral dip transformations for Illumina MiSeq (excluding 28 wildtype references), and transformed with a CRISPR/Cas9 construct harbouring a quadruple *pU6‐26::sgRNA* (FAD2: #1‐#4; F5H: #5‐8; Com (combined *FAD2* and *F5H* gRNAs): #1–2 and #7–8; Table S1.1 and Figure 1) and different promoter‐*Cas9* combinations. Numbers of T1 and T2 samples for sequencing were grouped per construct

Construct	Number of T1 lines	Selected T1 line to produce T2	Number of T2 lines
pU6‐26::FAD2|pdCaMV35S::AtCas9	9	–	–
pU6‐26::F5H|pdCaMV35S::AtCas9	3	1	45
pU6‐26::Com|pdCaMV35S::AtCas9	10	1	45
pU6‐26::FAD2|pPcUbi4‐2::AtCas9	17	–	–
pU6‐26::F5H|pPcUbi4‐2::AtCas9	20	–	–
pU6‐26::Com|pPcUbi4‐2::AtCas9	30	3	105
pU6‐26::FAD2|pRPS5A::AtCas9	6	2	76
pU6‐26::F5H|pRPS5A::AtCas9	16	3	145
pU6‐26::Com|pRPS5A::AtCas9	24	4	165
Total	135	14	581

–: no T1 or T2 plants selected for this case.

### Bioinformatics‐based mutation detection

Illumina MiSeq raw sequencing data were delivered as 192 sets of fastq files with forward and reverse reads (one per amplicon pool). Fastq files were sorted according to barcode (i.e. per T1 or T2 genotype) using FLEXBAR, giving nine sets of R1 and R2 fastq files (one set per barcode and one set for the cases the barcode could not be assigned) for each of the 192 pooled samples (Dodt *et al*., 2012; Roehr *et al*., 2017). Next, for each genotype, the sequence reads were mapped to the *FAD2* and *F5H* loci reference sequence using BBMap which can also map sequences with large indels which were expected with the multiple sgRNAs we used. Mapping results were stored in bam format (Bushnell, 2014; Li, 2011; Li *et al*., 2009). Variant calling was done using GATK4 MuTect2 and stored in VCF format (Cibulskis *et al*., 2013). Finally, Linux scripts (Appendix S1: .sh and .py files) were developed to detect somatic and germline CRISPR/Cas9‐induced mutations. This script allowed the identification of the most abundant mutation(s) but also rare mutations for each locus and CRISPR/Cas9 site.

The proportion of mutated loci and the mutant allele frequencies in targeted loci were determined from the frequencies in the sequence reads. For each mutated position in each locus, the two mutant alleles with the highest allele frequency were determined. These mutations could be either germline mutations or new somatic mutations. The position of mutated sites, indel length and mutation types were analyzed for the different sgRNA combinations. For T2 plants, the homozygosity or heterozygosity of germline mutations was determined for each of the three *FAD2* or *F5H* loci. A homozygous single allele germline mutant was scored if the highest frequency of a mutant allele was at least 0.85 and a biallelic full mutation was scored if the sum of the two highest mutant allele frequencies was at least 0.85. If only one mutant allele had a frequency higher than 0.4 but lower than 0.85, it was considered a heterozygous mutant/wildtype. Mutant alleles with frequencies lower than 0.4 were regarded as somatic mutations. An additional criterion was applied to validate that indeed the mutations were germline mutations: there should be no more than 5% new mutations at an already fully mutated sgRNA site. Such mutations would be somatic mutations that would contradict the supposition of a fully mutated (either homozygous or biallelic) sgRNA site. The threshold of 5% was used as some contaminant reads could be present in the pool of reads for a mutant (due to strand switches in the multiplexed PCR or due to barcode sorting errors). Out of 135 T1 plants, a set of 14 T1 plants with high a frequency of (somatic) mutations was selected to produce a T2 generation (581 plants), and a set of 31 T2 plants with germline indels in some or all *FAD2* and/or *F5H* loci/alleles was selected for production of T3 seeds. For the set of 31 selected T2 plants for T3 seed analysis, next to the assessment of the germline mutant allele count, also the total mutant allele frequency (germline plus somatic mutations) was determined by assessing the vcf‐files from Mutect2 combined with inspection of the reads in IGV to determine the frequency of mutant type reads (containing indels) as a fraction of the total number of reads (mutant + fully wildtype).

### Phenotyping

#### FA profiles in T2 leaves

The effect of *FAD2* mutations on FA profiles was checked in single leaves from 400 T2 plants (originating from 10 T1 plants) and 28 wildtype (WT) plants. C18:1 content compared to total FA methyl esters (FAME) content was determined by gas chromatography‐based quantification of FAME (GC‐FAME) similar to other studies in camelina (Li *et al*., 2006; Morineau *et al*., 2017). Lyophilized leaves were weighed, vortexed in 300 μL hexane and 50 μL 5 M KOH/MeOH, incubated in a heating block at 60 °C and 800 rpm for 5 min, cooled down to room temperature, and centrifuged in a tabletop centrifuge at 16 000 x ***g*** for 5 min. The hexane phases were loaded into a GC file with a glass insert and 5 μL injection volumes were quantified for FA using a GC flame ionization detector (GC‐FID).

#### Sinapine and FA profiles in T3 single seeds

The effect of *FAD2* and *F5H* mutations on FA and sinapine profiles was checked in single seeds: 150 T3 seeds originating from 15 T2 plants carrying mutations in both *FAD2* and *F5H*, 50 T3 seeds originating from 5 T2 plants carrying *FAD2* mutations, and 100 T3 seeds originating from 10 T2 plants carrying only *F5H* mutations.

The GC‐FAME method was the same as for leaves (but with three cycles of hexane extraction with 500 μL hexane with 0.26 mM glyceryl triheptadecanoate as internal standard and twice 250 μL hexane) to obtain a seed oil and a seed meal fraction after centrifuging. To the seed oil fraction, 200 μL 5 M KOH/MeOH was added. FA were quantified using GC‐FID. The seed meal samples were dried overnight at 103 °C, vortexed in 500 μL 70% MeOH, incubated in a heating block at 75 °C and 800 rpm for 20 min, cooled down to room temperature, and centrifuged at 13 000 rpm for 5 min. Supernatants were transferred to Eppendorf tubes. MeOH extractions were repeated twice with 250 μL 70% MeOH. The extract was filtered through a PTFE syringe filter (diameter 4 mm, pore size 0.45 μm), and the filtrate was transferred to glass screw cap vials with slitted PTFE septa caps. Sinapine content was quantified using a UPLC‐photodiode array (UPLC‐PDA) with a calibration curve ranging from 0 to 0.1 mg/mL sinapine thiocyanate.

#### Lignin profiles in T2 stems

*F5H* mutation effects on lignin profiles were checked in eleven air‐dried T2 stems (originating from five T1 plants) with three biological replicates and four WT samples. Monolignol composition was determined based on the protocol from Mokochinski *et al*., 2015 with adaptations (de Ascensao and Dubery, 2003; Jung and Shalita‐Jones, 1990; Mokochinski *et al*., 2015). Monolignols were obtained after hydrolysis from 100 mg air‐dried samples using 2 mL 4 M NaOH at 121 °C for 4 h. After cooling to room temperature, 6 mL MQ was added to the samples and ≥ 1 mL of each diluted solution was filtered through a ⌀4 mm 0.45 μm PTFE syringe filter. Subsequently, syringyl (S), p‐hydroxyphenyl (H) and guaiacyl (G) monolignols in 0.5 μL injection volumes were quantified using UPLC‐PDA and a standards calibration curve ranging from 0.005 to 0.1 mg/mL: 4‐hydroxybenzaldehyde (H′), vanillin (G′) and syringaldehyde (S′) in MQ.

### Statistics

After performing ANOVA, Fisher's protected LSDs were used to compare means of wildtypes and mutants.

## Results

### Combining *pRPS5A::AtCas9* and *pU6‐26::sgRNA* and using floral dip transformation is the most effective method for targeted mutagenesis in camelina

**Figure 2:**
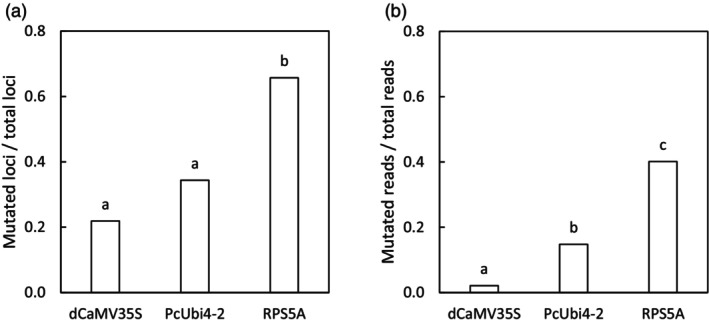
Comparisons of the ratio of mutated loci compared to the total amount of targeted loci (a) and mutated reads at target sites compared to the total amount of reads at mutated sites per targeted locus (b) between promoter‐*Cas9* combinations in T1 plants. Within each plot, significant differences between groups are indicated with different letters (*P* < 0.001).

Illumina MiSeq sequences of *F5H* and *FAD2* amplicons from leaf DNA samples of 135 T1 camelina plants with sgRNA under the *pU6‐26* promoter and *AtCas9* under three different promoters, *pdCaMV35S, pPcUbi4‐2 and pRPS5A* showed that the three promoters used, differed widely in mutation efficiency (Figure 2). *Cas9* expressed under *pRPS5A* was more effective than *pPcUbi4‐2* and *pdCaMV35S* (*P* < 0.001) measured by the proportion of mutated loci in all targeted loci and the frequency of mutant reads in all reads. The proportion of mutated loci with *pU6‐26::sgRNA and pRPS5A::AtCas9* was 66% on average and the frequency of mutant sequence reads was 40%. This includes both germline mutations induced already in the seeds growing on the T0 plants and novel somatic mutations that were induced in the T1 plants. Remarkably, in initial experiments with *pU6‐1::sgRNA* and pd*CaMV35S*Cas9, in 192 T1 and 576 T2 plants no mutations were detected at all, indicating that pU6‐1 is not functional in driving sgRNA expression in the floral dip transformation system used, and choosing a highly effective promoter like *pU6‐26* is essential.

### The CRISPR/Cas9 systems generated a wide variety of T1 and T2 mutants

Except for the sgRNA #6 target site, mutations occurred at all target sites. The highest frequency of mutations in T1 was found at the site of sgRNA #8 at 80% of all mutated *F5H* sites and at the sites of sgRNAs #1/#2/#3 (*FAD2*) each at ~30%, while sgRNA #4 was less effective and sgRNA #6 (with a design error) was not effective at all. The mutations in T1 were all indels: most were small 1 bp insertions (33% of all indels) or small deletions of 1–5 bp (40%). At target sites also frequently larger deletions (up to 30 bp; 16% of all indels) and larger insertions (up to 33 bp; in 2.7% of cases) were found. In about 9% of mutants, very large deletions of 31 to 290 bp were found between two sgRNAs targeting the same locus.

**Figure 3:**
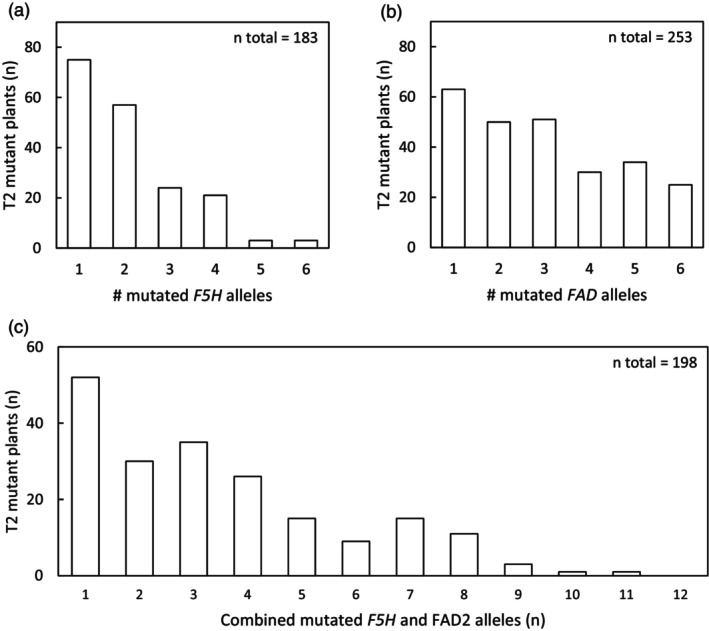
Distribution of T2 camelina mutants with varying numbers of mutated *F5H* alleles (a), mutated *FAD2* alleles (b), and combined mutated *F5H* and *FAD2* alleles (c). a and b include both T2 mutants with *F5H* or *FAD2* and combined CRISPR/Cas9 targets. The number of mutated alleles is determined by assuming a locus has a homozygous mutation or a biallelic full mutation when the total mutant allele frequency of one or two mutant haplotypes for a locus/sgRNA position >0.85 (mutant allele count = 2) and heterozygous with an allele frequency >0.4 (mutant allele count = 1).

T1 plants were selected for selfing based on the frequency of mutations detected in leaves (mostly somatic mutations). The total mutant allele frequency in the leaves of flowering T2 plants was used to assess the extent of Cas9‐induced mutations in the target loci. This total mutant allele frequency shows the efficiency of the Cas9 and chosen sgRNA sequences, but is higher than the germline mutant allele frequency as the CRISPR/Cas9 system was not segregated out and was still active. Germline mutant allele counts were based on the criteria mentioned in the Materials and Methods section (allele frequency of homozygous mutant alleles or the sum of the frequency of two mutant alleles in case of biallelic full mutants >0.85 and no somatic mutations at site already fully mutated in the germline). In the set of 581 T2 offspring plants originating from 14 selected T1 plants, 183 had at least one mutation in one or more of the three *F5H* loci and 253 had at least one mutated allele in one of the three *FAD2* loci (Figure 3). It is expected that to obtain a full knockout in the three genes of either *F5H* or *FAD2*, independent mutations are needed in all alleles (assuming that all alleles are functional). In the set of 505 T2 plants with sgRNAs targeting *F5H*, 10 T2 plants with independent mutations in each of the three *F5H* loci were found (2%), and in the 371 T2 plants with sgRNAs targeting *FAD2*, 87 T2 plants with a mutated allele in each of the three *FAD2* loci were found (23%). Twenty‐five T2 mutants were homozygous mutant or biallelic full mutant for all three *FAD2* loci and three were homozygous mutants (or fully biallelic mutant) for all three *F5H* loci. In the set of 335 T2 plants with sgRNAs targeting both *F5H* and *FAD2*, only one T2 plant (#28, Table 2) had germline mutations in all six *F5H* and *FAD2* loci. In conclusion, the multiplexing system targeting three loci of *FAD2* and three loci of *F5H* simultaneously with for each gene group two sgRNAs was successful in generating mutant alleles at all six loci in the T2 generation, but a large number of T1 and T2 offspring lines had to be screened to achieve this.

**Table 2:** T2 camelina mutants including their genotypes and phenotypes, as well as the phenotypes of corresponding T3 seeds. Sinapine expressed in g per kg total seed dry weight (1 g sinapine/kg = 3.22 mmol/kg = 3.22 μmol/g)

#T2 plant	Germline mutant allele count		Total mutant allele frequency		T2/T3 phenotypes						
	F5H	FAD2	F5H	FAD2	Growth habit T2 plant	C18:1 T2 leaf (%FA)	C18:1 T3 seeds (%FA)	C20:1 T3 seeds (%FA)	PUFA T3 seeds (%FA)	Sinapine T3 seeds (g/kg)	Lignin S/(H+G+S) ratio T2 stems (%)
*FAD2* mutants											
1		5		1.00	Dwarf	23	52	15	11		
2		6		1.00	Dwarf	18	58	23	5.2		
3		5		0.92	Dwarf	23	58	23	5.0		
4		4		0.69	Delayed	9	29	21	33		
5		5		0.92	Dwarf	23	*	*	*		
6		3		0.40	Normal	11	23	16	38		
*F5H* mutants											
7	5		0.96		Normal					0.24	4
8	6		0.99		Normal					0.21	0
9	6		1.00		Normal					0.03	n.d.
10	6		0.98		Normal					0.01	0
11	6		1.00		Normal					0.16	n.d.
12	6		0.99		Normal					0	0
13	4		0.99		Normal					0.08	n.d.
14	4		0.86		Normal					0.52	n.d.
15	3		0.73		Normal					0	n.d.
16	2		0.79		Normal					1.14	12
Combined *F5H*/*FAD2* mutants											
17	4	5	0.97	1.00	Normal	18	49	19	12	0.78	0
18	4	6	0.98	1.00	Delayed	21	54	11	11	0.65	6
19	3	2	0.73	0.96	Normal	20	23	21	38	2.30	n.d
20	4	4	1.00	0.93	Delayed	19	52	21	10	0.24	n.d
21	5	3	1.00	0.98	Normal	18	49	24	13	0.35	0
22	2	6	0.94	0.99	Normal	15	53	22	8.5	0.26	n.d
23	5	5	1.00	0.99	Dwarf	21	51	12	13	0.14	n.d
24	1	0	0.57	0.69	Normal	13	26	21	35	2.27	n.d
25	4	2	0.94	0.97	Delayed	22	54	14	10	1.13	n.d
26	2	2	0.40	0.34	Normal	11	24	12	40	1.29	n.d
27	2	0	0.57	0.55	Normal	12	13	18	48	1.57	n.d
28	6	6	0.99	0.99	Dwarf	20	56	14	9.1	0.10	0
29	5	1	0.98	0.99	Normal	23	50	19	12	0.31	0
30	3	3	0.59	0.72	Normal	11	17	15	44	2.30	17
31	2	2	0.58	0.65	Normal	13	29	21	30	0.91	n.d
WT	0	0	0	0	Normal	3.4	8.7	12	53	2.38	47

WT = wildtype. Leaf phenotypes were based on single leaves (mutant T2) and 28 leaves for WT. Seed phenotypes were based on the average of ten seeds (T3) or 26 seeds (WT). Stem phenotypes were based on the average of three biological replicates (T2) or four stems (WT). The overall best‐performing lines without T2 dwarf phenotypes are T2 plants #18 and #22. n.d.: not determined; *: insufficient number of seeds. Standard errors of means: C18:1 in WT leaves: 0.0042, C18:1 in seeds: 1.2, C20:1 in seeds: 0.56, PUFA in seeds: 1.1, sinapine in seeds: 0.195 and Lignin S/(S+H+G) in stems: 0.0014. Full details on germline mutant allele count per locus, total mutant allele frequency and type of mutations are described in Dataset S2, Table 2.1.

### Selected camelina *F5H* and *FAD2* mutants show altered FA, sinapine and lignin profiles

As an effect of mutations in *F5H*, the sinapine content in the seed was sharply reduced and in some even to undetectable levels. Also, the lignin syringyl monolignol content in T2 stem lignin of *F5H* mutants was drastically reduced (down to undetectable) in favour of G‐monolignol (Table 2). T2 plants with *FAD2* mutations had a higher C18:1 content in leaf tissue (23% of total FA) than WT (<4%). T3 seeds of these mutants showed a C18:1 content of 58% in the FA of seed oil compared to less than 9% in WT (Table 2). A high correlation existed between the C18:1 content in T2 leaf and T3 seeds from the same plant (*R* ^2^ = 0.65; Dataset S2, Table S2.2). The content of C20:1 in mutant T3 seeds was also increased up to 24% of total FA contents, and PUFA content (C18:2 and C18:3) was decreased down to 5% in some mutants.

### Effects of *F5H* and *FAD2* germline mutant gene dosage and total mutant allele frequency

**Figure 4:**
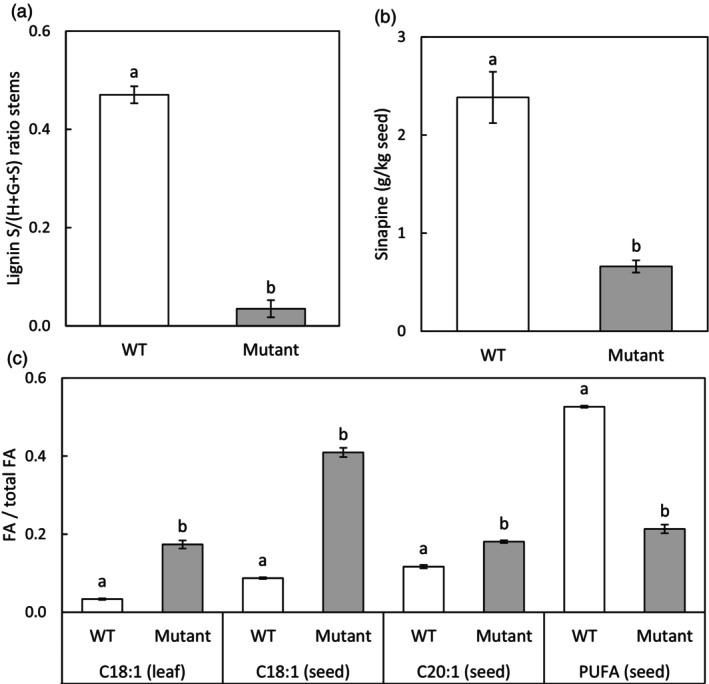
Phenotypic comparisons between WT (white bars) and genotypically selected T2 mutant plants and corresponding T3 seeds (grey bars) from Table 2. (a) *F5H* T2 mutants (stem); (b) *F5H* T3 mutants (seed); (c) *FAD2* T2 mutants (leaf) and T3 mutants (seed). Phenotypes were highly significantly different between mutants and WT, indicated by unique letters (*P* < 0.001).

Mutants in *F5H* had a 70% lower sinapine content and a 90% lower S/G ratio averaged over all mutants irrespective of the number of alleles mutated. All mutants in *FAD2* taken together showed a sharp increase in mono‐unsaturated FA and a decrease in poly‐unsaturated fatty acids (Figure 4). Also, C18:1 content in leaves (showing the reverse trend compared to PUFA) correlated highly to the total mutant allele count (Dataset S2, Table S2.2) as also shown by others (Morineau *et al*., 2017).

**Figure 5:**
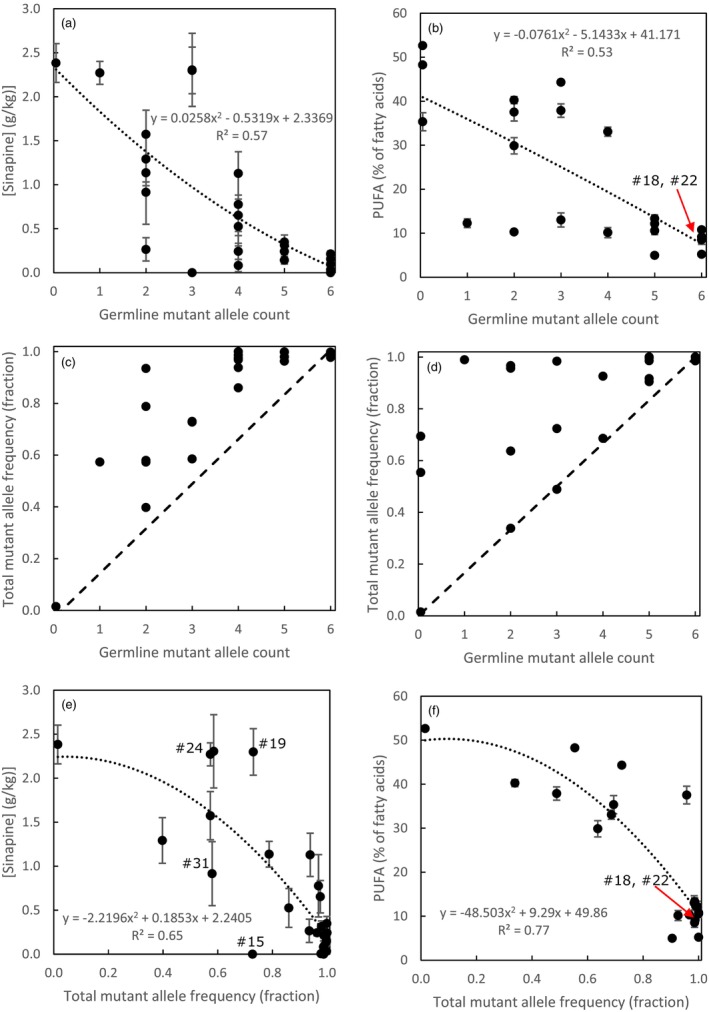
Relation between the mutant allele counts in the germline in the three *F5H* genes on sinapine concentration in the seed (g sinapine per kg seed dry weight) (a) and in the three *FAD2* genes on PUFA % of total fatty acid contents in seed oil (b). Relation between total mutant allele frequency (both germline and somatic mutations in the sampled leaf tissue) in the three genes of *F5H* (c) and in the three genes of *FAD2* (d) and the germline mutant allele count in these genes. The dashed line indicates the mutant allele frequency without somatic mutations. Relation between the total (germline and somatic) mutant allele frequency in the three genes of *F5H* and sinapine concentration in the seed (e) and in the three genes of *FAD2* and PUFA % of total fatty acid contents in seed oil (f). Means are shown for all 30 mutant T2 plants, in the case of sinapine of 10 replicate seeds, and in the case of PUFA of 30 replicate seeds. Error bars indicate +/‐ the standard error of the mean. #18 etcetera indicate the T2 plant number in Table 2.

In general, the effects increased monotonously with the number of mutated alleles in T2 plants that were inherited from the T1 plants (the germline mutations). Since the CRISPR/Cas9 system was not segregated out, new somatic mutations still occurred in the T2 plants, making the total mutant allele frequency much higher than the mutant allele frequency in the germline. For both *F5H* and *FAD2*, the decline in, respectively, sinapine and PUFA is more closely related to the total mutant allele frequency than the count of mutant alleles inherited in the T2 plants from the T1 generation (Figure 5). Plant #15 showed a strong decrease to an undetectable amount of sinapine, while the mutated allele count in the germline was only 4 and the total mutant allele frequency was only 0.73. This can only be explained when the particular mutation(s) would exert a dominant suppression also on the non‐mutated genes.

### Pleiotropic dwarfism with full knock‐out of *FAD2* loci: not with all mutants

**Figure 6:**
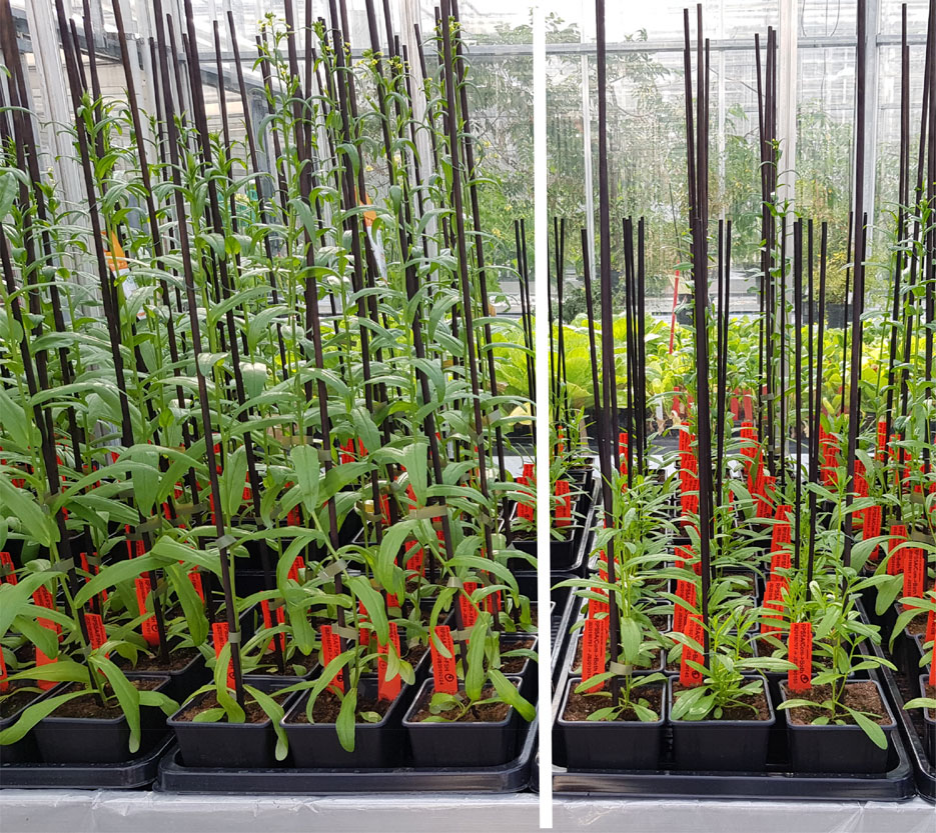
Mutants with three fully mutated *FAD2* loci with normally growing plants (left of the white bar) and other mutants with three fully mutated *FAD2* loci with dwarfed plants (right of the white bar).

In our study, five out of 21 T2 *FAD2* mutants (mostly with 5 or 6 of the *FAD2* alleles mutated) showed low PUFA but also a dwarf phenotype (Figure 6). Yet, not all our T2 *FAD2* mutants showed dwarfism. Some *FAD2* mutants showed a delayed growth and development only but eventually developed into normal plants with wildtype phenotype and wildtype seed yield. Two T2 plants (#18 and #22 in Table 2) with full germline mutations in all three *FAD2* genes had highly reduced PUFA contents but were not dwarfed and had good seed yield.

The germline mutations in the *FAD2* genes in plants #18 and #22 consisted of various homozygous deletions and biallelic full mutations (Table 3). Mutations occurred at single sgRNA sites (very often a 1 nt insertion of an A or T), but also simultaneous Cas9 action occurred at two sgRNA sites in which cases large deletions regularly occurred (for *FAD2* genes often 107 nt deletions). In few cases, triplet deletions or insertions occurred, for example, in plant #18 in *FAD2‐A*, but these mutations were always accompanied by a non‐triplet insertion or deletion at another sgRNA site. All *FAD2* loci in plants #18 and #22, therefore, had frameshift mutations. If triplet insertions or deletions are desired, it would be best to use only one sgRNA, so a triplet insertion or deletion yields a non‐frameshift mutation that cannot be disturbed by frameshift mutations at other sites. For plant #22 the selfed offspring was sown and the T3 plants still showed normal development in 66% of the cases, but 33% was dwarfed, probably because of new, additional germline mutations at sgRNA target sites not yet mutated in the T2.

**Table 3:** Type of mutations in the two non‐dwarfed full germline mutants T2 plants #18 and #22

Locus	Zygosity		Mutation
Plant #18			
FAD2‐A	BI	allele 1	1 nt ins (T) @ 1562 + 3 nt del (‐TCA) @ 1669
		allele 2	WT @ 1562 + 1 nt ins (A) @ 1669
FAD2‐B	HOM		107 nt del @ 1553
FAD2‐C	BI	allele 1	1 nt ins (A) @ 1537
		allele 2	107 nt del @ 1537
Plant #22			
FAD2‐A	BI	allele 1	107 nt del @ 1563
		allele 2	1 nt ins (T) @ 1669
FAD2‐B	BI	allele 1	1 nt ins (T) @ 1537 + complex mutation @ 1641
		allele 2	107 nt del @ 1537
FAD2‐C	HOM		11 nt del @ 1652

BI = biallelic full mutant, HOM = homozygous mutant, WT = wildtype. E.g. @ 1562 means the position at which the mutation in that locus starts with coordinates in the coding sequence (see Supplemental information Data S1 for the fasta file of the coding sequences).

## Discussion

### *pRPS5A::AtCas9* and *pU6‐26::sgRNA* are optimal combinations for CRISPR/Cas9 activity in camelina upon floral dip transformation

In this study, the *RPS5A* promotor to drive *Cas9* expression (*pRPS5A::AtCas9*) resulted in the highest CRISPR/Cas9 mutation frequency in leaves of T1 plants. This was expressed as a fraction of mutated reads per total number of reads (Figure 2B) and as the number of loci with mutated rates as a fraction of the total number of loci targeted (Figure 2B). The latter is higher as also with a low frequency of mutated reads for a locus, the locus counts as mutated in the T1 leaves. *pPcUbi4‐2::AtCas9* was only half as effective and *pdCaMV35S::AtCas9* resulted in the lowest mutation efficiency, which is in line with previous CRISPR/Cas9 studies in camelina and in the genetically very closely related *Arabidopsis* in which floral dip transformation was used (Fauser *et al*., 2012; Feng *et al*., 2013; Jiang *et al*., 2017; Lynagh *et al*., 2018; Tsutsui and Higashiyama, 2017; Wang *et al*., 2015; Wolter *et al*., 2018; Yan *et al*., 2016). Overall, the high mutation efficiency with *pRPS5A::AtCas9* in camelina was most likely the result of a high constitutive expression throughout all developmental tissues including egg cells, zygotes, early embryos and meristematic cells, whereas the low mutation efficiency with *pdCaMV35S::AtCas9* was most likely the result of limited expression until late embryonic development, while female reproductive tissues are the primary target during floral dip transformation (Desfeux *et al*., 2000; Lynagh *et al*., 2018; Sunilkumar *et al*., 2002; Tsutsui and Higashiyama, 2017; Weijers *et al*., 2001). Similarly, the high mutation efficiency with *pU6‐26::sgRNA* compared to the absence of mutants *with pU6‐1::sgRNA* in our camelina study was most likely the result of different sgRNA expression levels in egg cells, zygotes or early embryos and meristematic cells. A previous study in *Arabidopsis U6* genes showed that *U6‐26* had a higher expression than *U6‐1* and all other tested *U6* genes in all tested tissues, including leaf and stem tissue, but also in flower and silique tissue (X. Li *et al*., 2007). The true potential of using *pRPS5A::AtCas9* combined with *pU6‐26::sgRNA* in camelina upon floral dip transformation is reflected by our second‐generation mutants (Figure 3): 10% of the T2 mutants (with *FAD2* targets and mostly *pRPS5A::AtCas9*) showed fixed mutations in all six *FAD2* alleles, and 15% T2 plants had at least seven mutated *FAD2* and *F5H* alleles, including one *pU6‐26::sgRNA|pRPS5A::Cas9* mutant with eleven mutated alleles.

### *F5H* mutants yield seeds with zero sinapine and stems with zero S‐lignin

In our study, it was evident that we have obtained T3 camelina mutant seeds (from T2 plants) with *F5H* mutations that had significantly decreased sinapine contents compared to WT, and T2 mutant plants with significantly decreased lignin S/(H+G+S) ratios. On average sinapine content in mutant seeds went down from 2.4 to 0.7 g/kg seed with T3 lines going down to zero sinapine in all seeds. Moreover, lignin S/(H+G+S) ratios sharply decreased from 47% to 4% in stems in seven out of eleven T2 lines containing zero S‐monolignol. Previous studies have shown the crucial role of *F5H* in S‐lignin biosynthesis in *Arabidopsis*, tobacco and poplar using *F5H* overexpression and more recently in barrel clover using knockouts of *NST1* (i.e. a regulatory gene of *MYB58* that is an *F5H* transcription factor) (Franke *et al*., 2000; Meyer *et al*., 1998; Zhao *et al*., 2010). In rapeseed (*Brassica napus*) using *F5H*‐antisense the sinapine content in seeds was reduced by up to 40% or by up to 90% using combined *F5H*‐ and *SCT‐*antisense (Bhinu *et al*., 2009; Nair *et al*., 2000). In our study, we have confirmed the usefulness of CRISPR/Cas9 technology for targeted mutagenesis of *F5H* in polyploid camelina which resulted in a full loss of sinapine in seeds.

### *FAD2* mutants yield seeds with higher MUFA and decreased PUFA contents

Besides obtaining camelina seeds with significantly decreased sinapine contents, T3 camelina mutant seeds (from T2 plants) with *FAD2* mutations had both increased MUFA and decreased PUFA contents compared to WT (in % of total FA contents). These results were according to our expectations and in line with the findings of other *FAD2* knockout camelina studies (Jiang *et al*., 2017; Morineau *et al*., 2017). We have also obtained T3 seeds with combined *F5H* and *FAD2* mutations that showed expected effects of both types of mutations: lower sinapine contents from the *F5H* mutations and higher MUFA and lower PUFA contents caused by the *FAD2* mutations. On average, C18:1 content increased from <9% to 58% in T3 seeds and C20:1 content increased from 12% to 24%, while PUFA (C18:2 and C18:3) contents decreased from 53% to 5%. Furthermore, on average the total FA content per seed weight was increased by 40% compared to WT (*P* < 0.001, Data Set S1). This increased overall FA content per seed weight could have been caused by an increased seed weight of 76% on average compared to WT (*P* < 0.001, Data Set S1). To gain more insight into overall FA content per WT or mutant seed or whole plant, a follow‐up study should be set up that also takes into account the total seed set per plant, which has not been determined in our study. Nevertheless, we have obtained camelina seeds with significantly increased C18:1 and C20:1 contents and decreased PUFA contents.

### Germline mutant allele count or total mutant allele frequency

In this study, the Cas9/sgRNA system was not segregated out and still additional somatic mutations could arise in T2 plants and T3 plants. A clear effect of germline allele dosage for both gene groups was apparent, but it proved that the total mutant allele frequency (germline plus somatic as determined in leaves of flowering plants) showed a clearer monotonic effect on the phenotypic effects (lower sinapine or lower PUFA). Also, the correlation of the total mutant allele frequency with sinapine content (for *F5H*) and PUFA (for *FAD2*) was higher than the correlations with the germline mutant allele count (Figure 5). In some cases, the mutant allele frequency was close to 100% while the germline mutant allele count was only 2 or 3 (out of 6). When only the germline mutant allele count would be determined, in a case where the CRISPR/Cas9 system is still active, one could falsely conclude that also with a very low mutant allele count strong effects can be found, for example, very low sinapine in a seed with only a few mutated *F5H* alleles or few mutated *FAD2* alleles with very low PUFA. In fact, the strong effect is mediated through a large increase in the somatic mutation frequency. Still, in some cases, a total mutant allele frequency was only 0.75 while sinapine content in seed was reduced to non‐detectable (plant #15 in Figure 5e). Plant #15 has a biallelic full germline mutation in *F5H‐A*, a heterozygous mutant/wildtype genotype in *F5H‐B* and no germline mutation in *F5H‐C* and *F5H‐C* has only 17% somatic mutations in its leaf sample, so still functional *F5H‐C* could be available. Still, sinapine content was strongly reduced, which suggests that possibly one of the mutations had a dominant suppressive action, which can be mediated through for example truncated proteins interacting with the wildtype proteins produced by the non‐mutant genes (Meinke, 2013), as premature stop codons occurring with frameshifts caused by CRISPR/Cas9 do not always elicit nonsense‐mediated mRNA decay (NMD) (Tuladhar *et al*., 2019).

### Combined *FAD2* and *F5H* mutants with improved traits are sources for further breeding

We have successfully obtained a wide collection of genotypically and phenotypically different T3 camelina mutant seeds from an already wide collection of genotypically different T2 mutants (Data Set S1). These mutants form a unique source for in‐depth expression studies of *FAD2*/*F5H* in camelina, but form most importantly an excellent source for further camelina breeding and to fix specific *FAD2* and/or *F5H* mutated alleles and to remove T‐DNA sequences, if still present. Novel camelina cultivars can be obtained that yield both seeds with more MUFA and less sinapine while showing normal plant development and which have stems with optimal lignin compositions for multipurpose use, using all aboveground parts of the plant for bio‐based applications. These novel cultivars can then be used as starting materials for further improvements of seed properties. For example, in camelina, C18:1 content may be further increased in seeds by knocking out fatty acid elongase 1 (*FAE1*) which prevents the conversion of C18:1 into C20:1 (Ozseyhan *et al*., 2018). Combined *FAD2* and *FAE1* knockout mutants will expectedly lead to maximized C18:1 content in seeds, providing near‐homogeneous reaction mixtures for producing C10:0 from camelina seed oil. Moreover, the level of ANFs can be further reduced in seed meals by reducing glucosinolate contents, for example, by mutagenesis of glucosinolate transporters genes as shown in Brassica oilseeds (Nour‐Eldin *et al*., 2017).

### *FAD2* mutants with full germline mutations in *FAD2* and non‐dwarf phenotypes

Most of the lines with full germline mutations for the three *FAD2* loci showed dwarf growth and drastically less seeds. This phenomenon has also been observed in camelina (Jiang *et al*., 2017; Lee *et al*., 2021; Morineau *et al*., 2017). Here, two T2 plants (#18 and #22) with CRISPR/Cas9 germline mutations in both alleles of each of the three *FAD2* genes and also highly reduced PUFA, showed normal development which is unexpected as all *FAD2* genes had mutations.

Plant #18 had a delayed growth but reached the same final length as wildtype plants in the end. Plant #22 showed normal development while it had one of the highest MUFA and lowest PUFA contents in oilseeds (Table 2). Until now, only dwarfed phenotypes were found in previous studies with homozygous *FAD2* mutations in all three loci. This study shows it is possible to create homozygous or biallelic *FAD2* mutations in all three loci (impairing the function of FAD2) with low PUFA that do not show dwarfed phenotypes. As in our T2 plants CRISPR/Cas9 was still active, and not all sgRNA sites had been mutated already, further mutations could occur in the T2 plants.

The type of mutations in plants #18 and #22 does not differ clearly from any of the mutations in the other T2 plants with six germline mutated alleles in the three *FAD2* genes. The finding that these two T2 plants did not show dwarfed plants can, therefore, not be explained easily by the type of mutations. A further test on the T3 offspring of plant #22 showed that a large part of the T3 plants also did not show dwarfism (about 70%), but some T3 offspring did. This result would be obtained if one of the three *FAD2* genes was still heterozygous mutant/wildtype, however, the examination of the sequence reads indicated that in these two T2 plants, either fully homozygous mutations or full biallelic mutations were found in all loci (although not necessarily at all sgRNA sites). From the T2 to the T3 generation further germline mutations in the *FAD2* genes were possible at sgRNA sites that were not yet mutated as was also seen from the high frequency of somatic mutations that could be detected in many T2 plants. These new germline mutations that were inherited by the T3 plants might have induced the pleiotropic dwarfism that was not found in T2 plant #22 in which the *FAD2* genes were already fully mutated in the germline. Possibly, the new (unknown) mutations did have an effect on the pleiotropic response towards dwarfism. Depending on the type of mutation either truncated proteins can be produced or no protein at all when nonsense‐mediated mRNA decay (NMD) occurs, and this can potentially give different outcomes of phenotypes and pleiotropic effects caused by the targeted genes (Tuladhar *et al*., 2019).

## Conclusions

In this study, CRISPR/Cas9‐mediated genome editing in camelina was improved to such an extent that a high level of targeted mutagenesis can be achieved in multiple loci, already in T1 mutants. This highly effective system involved *Cas9* expression under the *RPS5A* promoter and sgRNA expression under the *U6‐26* promoter, the latter of which is critical for CRISPR/Cas9 activity upon floral dip transformation in camelina. To our knowledge, *pRPS5A* has not been used in other CRISPR/Cas9 studies with camelina and its use leads to very high transformation efficiency in camelina. Further, we have successfully used an improved Illumina MiSeq approach with custom barcodes and a custom script that allowed for a high‐throughput and extremely detailed screening of polyploid camelina mutants allowing determination of the total mutant allele frequency. Based on this, the total mutant allele frequency count of germline mutant alleles and the additional somatic mutation frequency were determined. With homozygous single mutant haplotype mutants, it proved important to screen for new mutations on sgRNA sites that were already fully mutated to assess if a true germline mutation had occurred. A low frequency of such apparently somatic mutation reads had to be allowed as a consequence of the high level of multiplexing the deep sequencing of such a large number of over 750 T2 mutants and required manual inspection as a quality control.

Ultimately, we have obtained a T3 camelina mutant seeds collection with a variety of *FAD2* and/or *F5H* mutated alleles. The combination of *FAD2* and *F5H* mutations is unique and has resulted in novel camelina lines with significant improvements in both seed and stem quality traits. Relative PUFA (C18:2 and C18:3) contents compared to total FA contents have been reduced from 53% in WT seeds to 5% in T3 seeds (from T2 plants), with individual T3 seeds containing down to 5% PUFA. Moreover, relative MUFA (C18:1 and C20:1) contents compared to total FA contents have been increased from 20% in WT seeds to 59% in T3 seeds, with individual T3 lines containing up to 81% MUFA. Moreover, we have demonstrated that it is possible to obtain a complete *FAD2* mutant with 75% MUFA and 8.5% PUFA contents in oilseeds without a dwarf plant phenotype, which has not yet been reported in other camelina studies. The camelina seed oil profile can be further improved by knocking out *FAE1* to reduce C20:1 contents in favour of more C18:1 contents. Sinapine contents have been significantly reduced in T3 seeds, with individual T3 seeds containing 0 mg sinapine per kg seed. The camelina seed feed value can be further improved by reducing glucosinolates contents. As an interesting side‐effect, lignin S/(H+G+S) ratios in T2 stems have been drastically reduced to zero or nearly zero which improves biomass hydrolizability for producing biofuels. In conclusion, we have obtained a unique source for in‐depth studies of *FAD2*/*F5H*, but above all an excellent putative source for further breeding in already significantly improved camelina lines.

## Supporting information


**Table S1.1.** Camelina FAD2 and F5H gene‐specific sgRNAs.
**Table S1.2**. FAD2 and F5H genes gene‐specific primers (shown without adapters and unique barcodes).

**Figure S1.** Illumina MiSeq Preparation Workflow based on the 16S Metagenomic Sequencing Library Preparation Guide (version #15044223 Rev. B, Illumina) with adaptations.

**Figure S2.** Mutant with three fully mutated FAD2 loci with normal (left) and other mutant with fully mutated FAD2 loci with dwarfed plants (right).

**Appendix S1.** Linux scripts to detect somatic and fixed CRISPR/Cas9‐induced mutations.

**Dataset S2.** Table S2.1 with the assessment of germline mutant allele count and somatic mutation frequencies, including the full details on the type of mutations in Table 2.
Table and Figure S2.2. FA content of T2 leaves (FA) content.
Dataset_S3.xlsx: FA content, sinapine content and lignin composition.

**Data S1.** Coding sequences of the target *F5H* and *FAD2* genes:

## Data Availability

Data are available upon request to the corresponding author.
