# Supplementary material for: Direct and indirect effects of multiplex genome editing of F5H and FAD2 in oil crop camelina
Source: Plant Biotechnol J. 2025 Jan 27;23(5):1399–412. doi: 10.1111/pbi.14593 (PMC12018816; doi:10.1111/pbi.14593)
Supplement: Supplementary file 1 — Table S1.1. Camelina FAD2 and F5H gene‐specific sgRNAs. Table S1.2. FAD2 and F5H genes gene‐specific primers (shown without adapters and unique barcodes). [file PBI-23-1399-s005.docx]

***Supplemental information on sgRNA and gene-specific primers used***

***Table S1.1****: Camelina FAD2 and F5H gene-specific sgRNAs.*

| *sgRNA* | *Target* | *sgRNA (target-specific spacer) sequence* |
| --- | --- | --- |
| sgRNA-#1 | *FAD2* | 5'-GAGCGTTTGAAACACTGCGG-3' |
| sgRNA-#2 | *FAD2* | 5'-GGTAAGAGAGAGGCTGAGGG-3' |
| sgRNA-#3 | *FAD2* | 5'-GGTGGCGACGTAGTAGAAGC-3' |
| sgRNA-#4 | *FAD2* | 5'-GCTGTGTCCTAACCGGTGTC-3' |
| sgRNA-#5 | *F5H* | 5'-GGGCTTGTGCCATCTCCGCA-3' |
| sgRNA-#6* | *F5H* | 5'-GCATAAACATATTGCCTATG-3' |
| sgRNA-#7 | *F5H* | 5'-GTATTTGACTTACGACCGAG-3' |
| sgRNA-#8 | *F5H* | 5'-GAGCTAGATTGGCTAAACCA-3' |

*N/A: RE not available. *: Incorrectly designed sgRNA.*

***Table S1.2****: FAD2 and F5H genes gene-specific primers (shown without adapters and unique barcodes).*

| *Gene* | *Forward (F) primer* | *Reverse (R) primer* |
| --- | --- | --- |
| *FAD2* | 5'-TGGGTGCAGGTGGAAGAATG-3' | 5’-GGACGAGAAGGAAGGAATGG-3' |
| *F5H* | 5'-CATCAYACRACGACGAAGGC-3' | 5'-GAGACCGACCGGACCATTTT-3' |
