# Supplementary material for: Direct and indirect effects of multiplex genome editing of F5H and FAD2 in oil crop camelina
Source: Plant Biotechnol J. 2025 Jan 27;23(5):1399–412. doi: 10.1111/pbi.14593 (PMC12018816; doi:10.1111/pbi.14593)
Supplement: Supplementary file 2 — Figure S1. Illumina MiSeq Preparation Workflow based on the 16S Metagenomic Sequencing Library Preparation Guide (version #15044223 Rev. B, Illumina) with adaptations. [file PBI-23-1399-s002.docx]

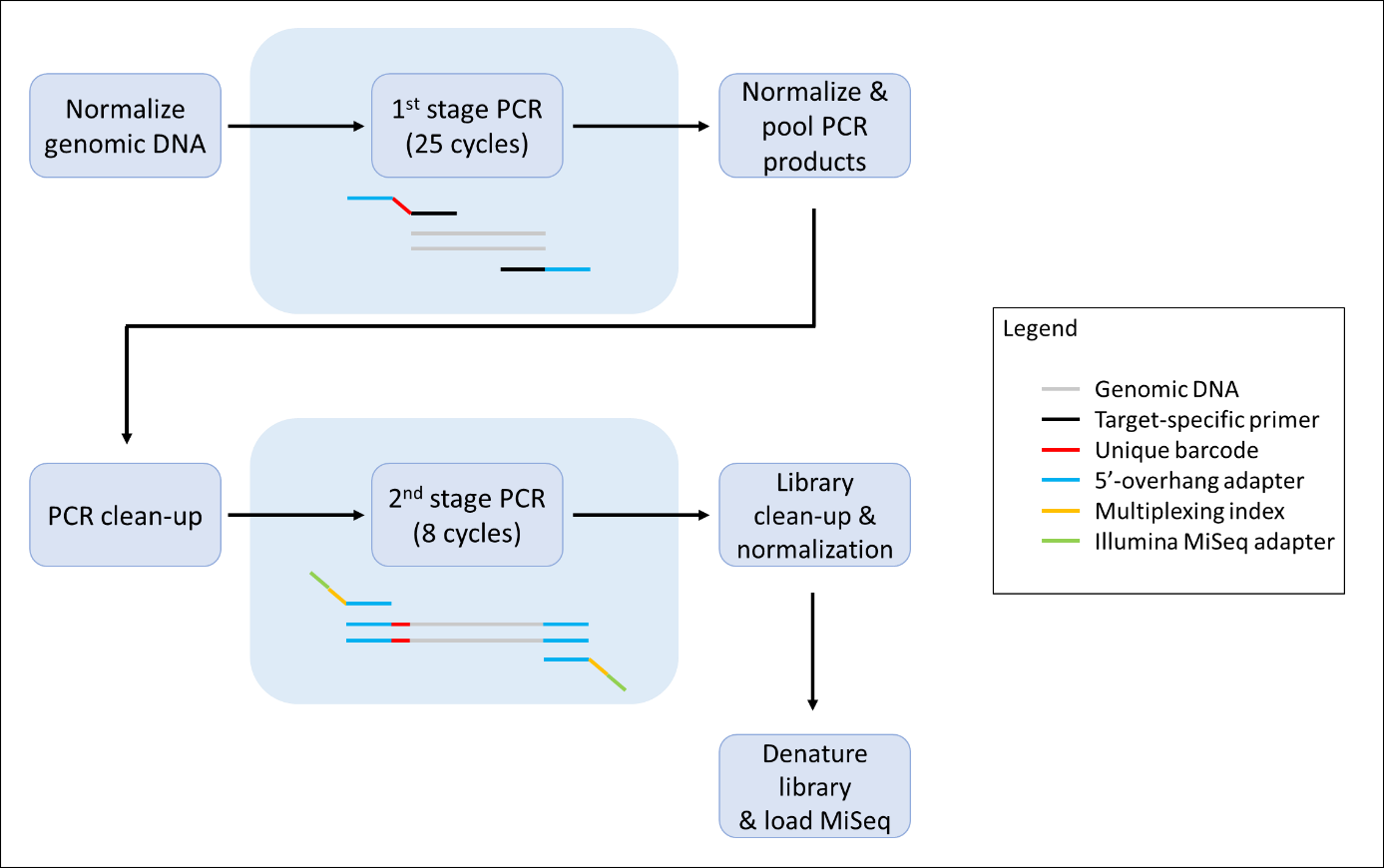


***Figure S1****: Illumina MiSeq Preparation Workflow based on the 16S Metagenomic Sequencing Library Preparation Guide (version #15044223 Rev. B, Illumina) with adaptations. Genomic DNA samples were normalized using a Qubit 2.0 Fluorometer (Invitrogen) and a 25-cycle “1^st^ Stage PCR” was run with FAD2 and F5H gene-specific primers, including adapters for “2^nd^ Stage PCR” and with 12.5 or 25 ng genomic DNA template respectively (depending on the primers efficiency). “1^st^ Stage PCR” products were quantified using gel electrophoresis and ImageJ (National Institutes of Health, MD, USA) based on the methods from Davarinejad (York University, ON, CA), and subsequently normalized by pooling in two 96-well plates*. Remaining steps, including a.o. an 8-cycle “2^nd^ Stage PCR” to add 96 indices per plate (for pooling) and Illumina sequencing adapters (for Illumina MiSeq), were carried out by the Bioscience department of Wageningen University & Research (The Netherlands).*

Example of ordered FAD2 forward primer for 1^st^ stage PCR:
**5’-TCGTCGGCAGCGTCAGATGTGTATAAGAGACAGATGACGTGGGTGCAGGTGGAAGAATG-3’**where blue represents the adapter, red the unique barcode, and black the target-specific sequence (as in **Figure S1**).

*Davarinejad, H. Quantifications of Western Blots with ImageJ. <http://www.yorku.ca/yisheng/Internal/Protocols/ImageJ.pdf> (2017).
