# Supplementary material for: Direct and indirect effects of multiplex genome editing of F5H and FAD2 in oil crop camelina
Source: Plant Biotechnol J. 2025 Jan 27;23(5):1399–412. doi: 10.1111/pbi.14593 (PMC12018816; doi:10.1111/pbi.14593)
Supplement: Supplementary file 3 — Figure S2. Mutant with three fully mutated FAD2 loci with normal (left) and other mutant with fully mutated FAD2 loci with dwarfed plants (right). [file PBI-23-1399-s001.docx]

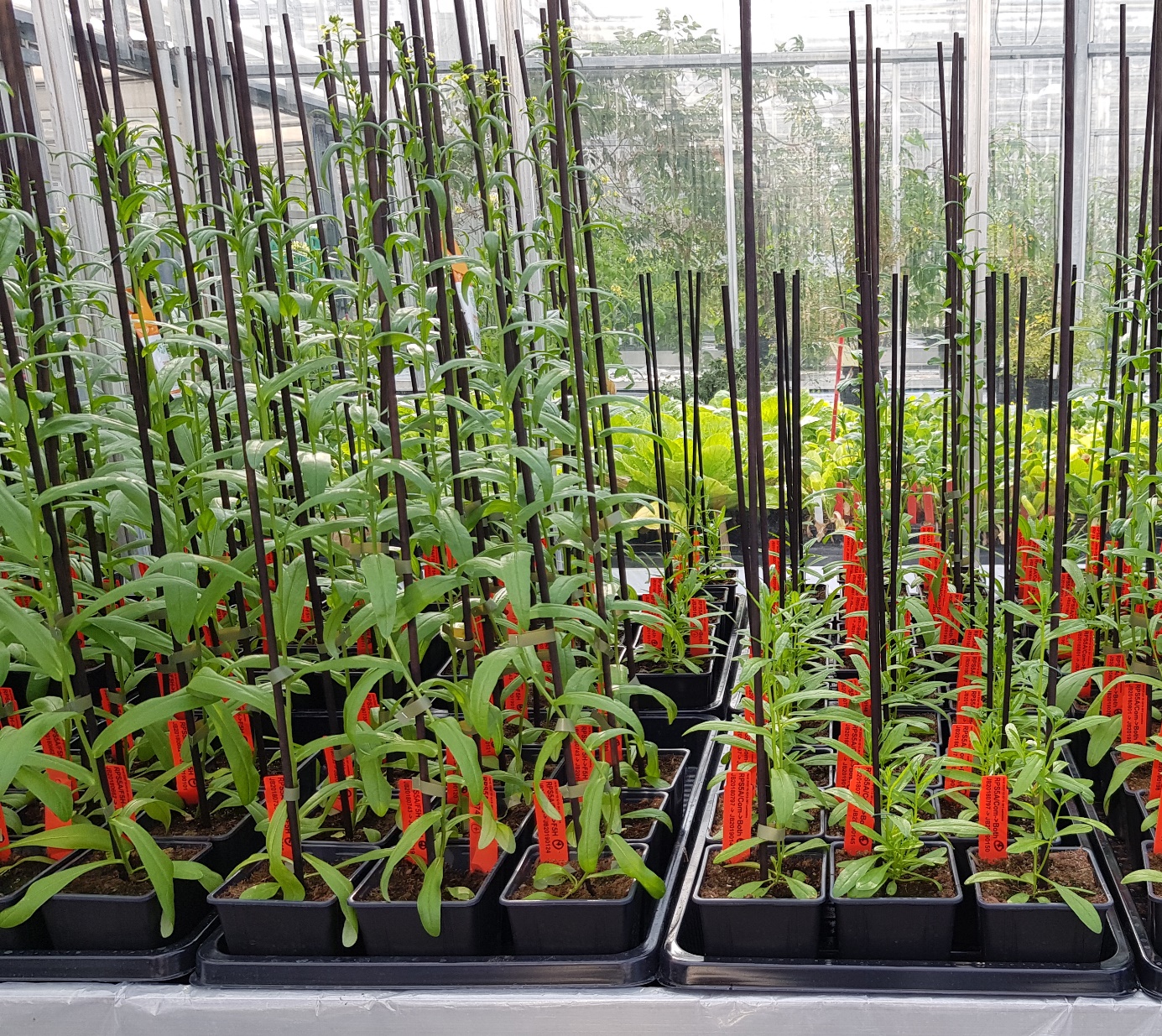


Fig. S2. Mutant with three fully mutated FAD2 loci with normal (left) and other mutant with fully mutated FAD2 loci with dwarfed plants (right)
